# Regulation of HIV-1 transcription in cells of the monocyte-macrophage lineage

**DOI:** 10.1186/1742-4690-6-118

**Published:** 2009-12-23

**Authors:** Evelyn M Kilareski, Sonia Shah, Michael R Nonnemacher, Brian Wigdahl

**Affiliations:** 1 Center for Molecular Virology and Translational Neuroscience, Institute for Molecular Medicine and Infectious Disease, Drexel University College of Medicine, 245 N 15th St, Philadelphia, Pennsylvania 19102, USA; 2 Center for Molecular Therapeutics and Resistance, Institute for Molecular Medicine and Infectious Disease, Drexel University College of Medicine, 245 N 15th St, Philadelphia, Pennsylvania 19102, USA; 3 Department of Microbiology and Immunology, Drexel University College of Medicine, 2900 Queen Lane, Philadelphia, Pennsylvania 19129, USA

## Abstract

Human immunodeficiency virus type 1 (HIV-1) has been shown to replicate productively in cells of the monocyte-macrophage lineage, although replication occurs to a lesser extent than in infected T cells. As cells of the monocyte-macrophage lineage become differentiated and activated and subsequently travel to a variety of end organs, they become a source of infectious virus and secreted viral proteins and cellular products that likely initiate pathological consequences in a number of organ systems. During this process, alterations in a number of signaling pathways, including the level and functional properties of many cellular transcription factors, alter the course of HIV-1 long terminal repeat (LTR)-directed gene expression. This process ultimately results in events that contribute to the pathogenesis of HIV-1 infection. First, increased transcription leads to the upregulation of infectious virus production, and the increased production of viral proteins (gp120, Tat, Nef, and Vpr), which have additional activities as extracellular proteins. Increased viral production and the presence of toxic proteins lead to enhanced deregulation of cellular functions increasing the production of toxic cellular proteins and metabolites and the resulting organ-specific pathologic consequences such as neuroAIDS. This article reviews the structural and functional features of the cis-acting elements upstream and downstream of the transcriptional start site in the retroviral LTR. It also includes a discussion of the regulation of the retroviral LTR in the monocyte-macrophage lineage during virus infection of the bone marrow, the peripheral blood, the lymphoid tissues, and end organs such as the brain. The impact of genetic variation on LTR-directed transcription during the course of retrovirus disease is also reviewed.

## Introduction

Approximately 33.2 million people are infected with the human immunodeficiency virus type 1 (HIV-1) worldwide, including 2.5 million people who were newly infected in 2007 [1]. Although fewer people are currently infected with HIV type 2 (HIV-2), this virus is spreading from its origin in West Africa to the Americas, Asia, and Europe [2] and reviewed in [3-5]). In addition to being the causative agent of the acquired immunodeficiency syndrome (AIDS), HIV-1 can cause neurological problems, ranging in severity from minor cognitive/motor dysfunction (MCMD) to HIV-1-associated dementia (HAD) (reviewed in [6-9]).

Cells of the monocyte-macrophage lineage play an important role in the transmission and pathogenesis of HIV [10-12]. When transmission occurs vaginally, rectally, or orally, the primary cells involved in the transmission event are dendritic cells [13]. However, during mucosal trauma, inflammation, and ulceration, the epithelial barrier may be disrupted and provide HIV with direct access to the mucosal microcirculation and/or provide directional signals to recruit highly susceptible, activated, inflammatory monocytes and T cells [14]. Circulating monocytes can also be infected and then migrate to peripheral tissues, including the brain [15,16], lung [17], lymphatic system [18], bone marrow [19,20], and kidney (reviewed in [21]). Infected monocytes can differentiate into monocyte-derived macrophages (MDMs) and may form a long-lived reservoir for the virus [22-25]. Additionally, MDMs can be infected after differentiation and are more susceptible to new infection in comparison to freshly isolated monocytes due to increased expression of the HIV co-receptor CCR5 [26]; however, this infection is limited, and the production of virus is hindered at many steps which will be discussed. Infected MDMs can seed the periphery with new infectious virus [20], directly transmit virus to T cells [27,28], release toxic viral proteins [29-31], and produce an altered array of cytokines and effector functions that contribute to HIV pathogenesis [32-35]. Additionally, infected monocyte progenitor cells can harbor virus in the bone marrow and seed the periphery with infected daughter cells. As these cells differentiate in the marrow and periphery, the levels of HIV-1 transcription may increase, resulting in the expression of toxic viral proteins and enhanced replication [36] and Alexaki, Shah, and Wigdahl, unpublished results). These cells can also cross the blood-brain barrier and deliver virus to the central nervous system.

Retroviral gene expression is regulated in a cell type- and differentiation-dependent manner by the binding of both host and viral proteins to the long terminal repeat (LTR), which serves as the viral promoter (reviewed in [37]). Host transcription factors such as the Sp family, nuclear factor kappa B (NF-κB) family, activator protein 1 (AP-1) proteins, nuclear factor of activated T cells (NFAT), and CCAAT enhancer binding protein (C/EBP) family members play key roles in the regulation of retroviral transcription by binding sites in the LTR that display different levels of sequence conservation. Viral proteins such as HIV Vpr and Tat also bind to the LTR to regulate transcription. Many of these host and viral proteins engage in extensive protein-protein interactions, leading to a complex system of transcriptional regulation. Adding to this complexity, the genomes of HIV-1, HIV-2, and simian immunodeficiency virus (SIV) accumulate a significant spectrum of genetic alterations as the virus replicates. When present in the LTR, these sequence alterations affect the ability of host and viral proteins to bind to their cognate binding sites and result in altered transcriptional and replication potential of the virus [38-46].

Regulation of HIV-1 transcription in cells of the monocyte-macrophage lineage varies considerably with the differentiation stage of the cell. Specifically, it has been observed that cyclin T1 expression in monocytes is controlled by differentiation. Cyclin T1 increases as cells of the monocyte-macrophage lineage differentiate [47]. This is important because cyclin T1 is one-half of the positive transcriptional elongation factor b (P-TEFb) complex necessary for the binding of Tat to TAR for the induction of HIV-1 transcription. Unstimulated peripheral monocytes and myeloid progenitor cells support low levels of viral replication and transcription in response to cellular activation [27,36,48-54], whereas differentiated MDMs have increased viral replication but either do not respond to [45] or downregulate HIV transcription [48,55] in response to cellular stimulation. During late-stage disease and AIDS, when CD4^+ ^T cells have largely been depleted, HIV-1-infected MDMs represent a greater component of the total infected cell population, and this pool of virus contributes significantly to the circulating levels of virus *in vivo *[56,57].

### Lentiviral LTR Structure

**Figure 1:**
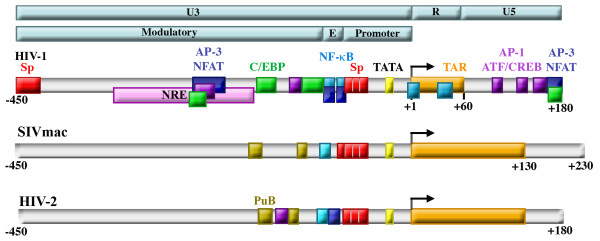
**Structure of retroviral LTRs**. Retroviral LTRs are divided into the U3, R, and U5 regions, and the U3 region is further divided into the Modulatory, Enhancer (E) and Promoter regions (top bars). HIV-1, HIV-2, and SIV all contain highly conserved promoters containing TATA boxes (yellow) and Sp factor binding sites (red) and enhancers (labeled E in light blue bar) containing NF-κB binding sites (blue). The R region of each contains a trans-acting responsive element (TAR) (orange) that forms an RNA stem loop structure upon transcription that binds to the viral protein Tat. A negative regulatory element (NRE, pink) was identified that was subsequently shown to serve as both activator and repressor by binding NFAT proteins (dark blue), AP-1 proteins (purple), and C/EBP factors (green). The modulatory regions of SIVmac and HIV-2 also contain purine box arrays (PuB, gold) and sites that bind members of the Ets family (teal).

Lentiviral LTRs are comprised of U5, R, and U3 regions. The U3 region is further divided into the core promoter, enhancer, and modulatory regions [37]. Lentiviral LTRs, HIV-1, SIV, and HIV-2, have closely related core promoters (Sp binding sites) and enhancer regions (NF-κB binding sites) (Fig. 1). These *cis*-acting elements allow for efficient replication in a variety of cell types and conditions that result in differential availability and activation state of transcription factors in the nucleus. However, the modulatory region is less closely related between lentiviral LTRs and contributes to the ability of the LTR to regulate transcription in various cell types and under various cellular conditions. These concepts are discussed below.

### Core promoter and enhancer regions: the interaction of Sp, NF-κB, and NFAT proteins

#### Sp factors

The core promoters of HIV-1, HIV-2, and SIV all contain a TATA box and multiple binding sites for the Sp family of transcription factors, and their enhancers all contain at least one binding site for NF-κB. The Sp and NF-κB factor binding sites in the core promoter play important cell type-specific roles in regulating transcription and replication. The promoter of HIV-1 contains three binding sites for Sp factors at -46 to -78 relative to the transcriptional start site (Fig. 1) [58]. Sp factors also regulate transcription by binding to positions +271 to +289 [59,60] and -421 to -451 [61] relative to the transcriptional start site. Sp family members include Sp1-4, as well as M1 and M2, truncated Sp3 proteins that result from alternative translational start sites within the transactivation domain [62-65]. All of the Sp proteins contain zinc finger DNA binding domains, and Sp1, 3, and 4 have similar, though not identical, affinities and specificities for GC-rich (GGGGCGGGGC) DNA [62,66,67]. Sp2 binds to GT-rich sequences (GGTGTGGGG) rather than to the GC-rich sequences that constitute the classical Sp binding sites [65]. Sp1 and Sp4 are transcriptional activators, whereas Sp3 has been classified as a repressor of HIV-1 transcription. By itself, Sp3 can weakly activate HIV-1 transcription; however, in the presence of the strong activator Sp1, it competes for binding to the LTR and inhibits activation by Sp1 [66,68,69]. In contrast, M1 and M2 have the Sp3 DNA binding domain but lack the transactivation domain and are true repressors of transcription in the absence or presence of other Sp family members [69]. In addition to repressing Sp-mediated transactivation, Sp3 represses LTR activation by the viral protein Tat [66]. Sp4 is expressed predominantly in the brain [62,70,71], providing an additional HIV-1 LTR transactivator to drive replication in this compartment. Unlike Sp1, Sp4 does not synergistically activate transcription in the presence of multiple Sp binding sites [71]. Consequently, the loss of one binding site due to genetic variation may have less of an effect in the brain than it would in other tissues, because the loss of function would not synergistically disrupt binding.

Genetic variation within the Sp sites is likely to play a role in HIV-1-associated disease progression. The NF-κB-proximal Sp site (site III) is much less conserved during the course of disease than Sp sites I and II [41] and Kilareski and Wigdahl, unpublished results). A C-to-T change at position 5 of Sp site III has been shown to correlate positively with HIV-1-associated disease progression, both in the periphery and in the brain [41]. This variant greatly reduces the affinity of this site for Sp factors, but greatly increases the response of viral replication to tumor necrosis factor α (TNFα) stimulation in peripheral blood mononuclear cells (Kilareski, Pirrone, and Wigdahl, unpublished observation). This finding is likely due to a loss of steric hindrance leading to an increase in NF-κB binding to its adjacent binding sites (Liu, Banerjee, and Wigdahl, unpublished observations). In the presence of Sp4 in the brain, one could speculate that this effect may be magnified, because Sp4 binding to sites I and II is not affected by the loss of Sp binding to site III, and the resulting stimulated LTR may have high levels of both Sp and NF-κB factors bound to their cognate sites.

Sp factors bound to the HIV-1 core promoter cooperate with the TATA binding protein and TATA binding protein-associated factors 110 and 55 to drive basal transcription [72-75]. They can also recruit P-TEFb to promote phosphorylation of RNA Pol II [76] and play an important role in remodeling chromatin to facilitate or inhibit transcription [77,78]. Histone deacetylases (HDACs) 1 and 2 are regulated through phosphorylation by protein kinase CK2. Sp1 and Sp3 can bind and recruit the phosphorylated HDACs to the LTR to repress LTR activity [79-81]. The repressor activity of Sp1 and Sp3 is regulated by the expression of CK2 [77].

The three Sp sites in the HIV-1 promoter have different affinities for Sp factors [39,40,58,82], and the affinity of Sp for LTR binding sites correlates with replication kinetics; faster viral replication is achieved when a higher affinity Sp binding site is in the NF-κB proximal site [39]. Interestingly, this might, at first glance, seem to contradict the fact presented above that Sp site III has increased genetic variation with the 5T variant (a low binding affinity site) correlating with disease progression, given traditionally low binding affinity correlates with decreased viral production. However, given that a decreased binding affinity has been shown to promote higher levels of NF-κB binding, this variation may actually provide an opportunity for increased replication (Kilareski and Wigdahl, unpublished observations). This suggests that genetic variations within these sites could have significant effects on the overall viral replication kinetics [41].

Expression patterns of the different Sp isoforms can modulate HIV-1 transcription in different cell types. As cells of the monocyte lineage differentiate, the ratio of Sp1 to Sp3 increases, resulting in increased HIV-1 transcription (McAllister and Wigdahl, unpublished observations). This process allows HIV to replicate at low levels, if at all, in circulating monocytes, and to evade the immune system until the cells are differentiated in peripheral tissues. The importance of the Sp sites also varies depending on the differentiation stage of the cell; in unstimulated monocytes, mutation of the Sp sites reduces LTR activity, whereas in MDMs, transcription of HIV and replication of SIVmac are abolished when these critical binding sites are knocked out [83-86].

**Figure 2:**
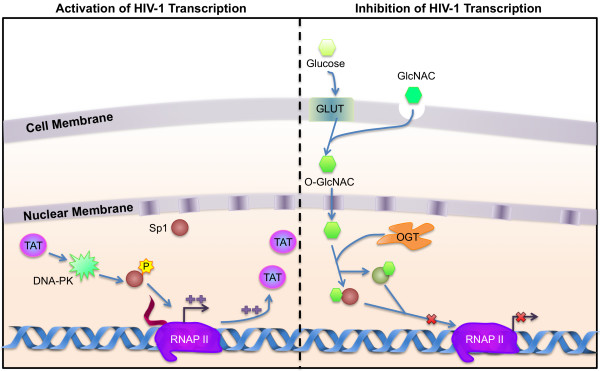
**Important Sp transcription factor signaling in monocyte-macropahges**. (a) Activation of HIV transcription by the interaction of viral protein Tat with DNA-dependent protein kinase (DNA-PK) results in the subsequent phosphorylation at Ser131 of Sp1. Phosphorylated Sp1 results in increased transcription of proviral DNA, resulting in an increase in Tat production, perpetuating the cycle. (b) Inhibition of HIV transcription involves O-linked N-acetylglucosamine (O-GlcNAc) transferase (OGT) catalyzing the addition of O-GlcNAC to Sp proteins which blocks their interaction with their binding sites on the LTR, resulting in an inhibition/reduction in HIV transcription.

DNA binding and transactivation activity of Sp factors are regulated both positively and negatively by phosphorylation and other post-translational modifications (reviewed in [87,88] and Fig. 2). Phosphorylation at Sp1 Ser131 by DNA-dependent protein kinase increases the affinity of the protein for DNA and also increases the ability of the protein to cooperate with the viral protein Tat to transactivate the LTR [89-92]. In contrast, O-linked N-acetylglucosaminylation (O-GLcNAc) of Sp1 inhibits HIV-1 replication [93]. Therefore, modulating O-GLcNAc of transcription factors may play a role in regulation of HIV-1 latency and activation, and may link glucose metabolism to HIV-1 replication.

#### NF-κB

**Figure 3:**
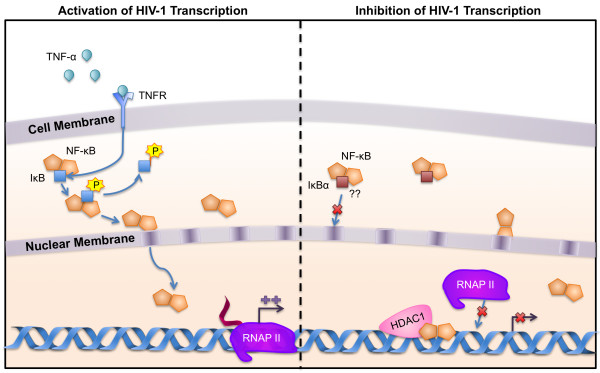
**Important NF-κB transcription factor signaling in monocyte-macrophages**. (a) Activation of HIV transcription: Translocation of NF-κB from the cytoplasm to the nucleus is controlled by association of IκB with the NF-κB hetero-/homo-dimer. Once IκB is phosphorylated, it relesases NF-κB which then translocates to the nucleus where it can bind the LTR and induce HIV transcription. (b) Inhibition of HIV transcription: In T cells, IκBα has been shown to contribute to lower levels of LTR transcription and potentially contribute to latency. It is postulated that a similar mechanism of action could be in place for cells of the monocyte-macrophage lineage. In addition, NF-κB's association with the histone deacetylase inhibitor HDAC1 results in constriction of the chromatin so that RNA polymerase does not have access to its target DNA.

NF-κB proteins have been shown to be one of the main modulators of the HIV-1 LTR in all cell types and a potential pathway for anti-HIV-1 therapies [94]. NF-κB proteins bind the enhancer at two sites located at nucleotide positions -81 to -91 and -95 to -104 relative to the transcriptional start site [95-97]. NF-κB is composed of heterodimers of five c-rel protein family members: p65/RelA, NF-κB1/p50, c-Rel, RelB, and NF-κB2/p52. Functional NF-κB in T cells is predominantly composed of p65 or c-Rel bound to p50 or p52, whereas in MDMs, Rel B replaces p65 [97-100]. In T cells and immature monocytes, NF-κB shuttles between the cytoplasm and the nucleus in response to cellular stimuli. In the cytoplasm, NF-κB is bound to inhibitor (IκB) proteins [101]. As a result of specific stimuli, IκB is phosphorylated and released from NF-κB; after release from the inhibitory complex, NF-κB translocates to the nucleus where it activates many host and viral genes through the initial recruitment of P-TEFb (Fig. 3) [101-103]. Interestingly, one of the IκB's, IκBα has been shown to play a role in shuttling of NF-κB from the nucleus and cytosol and in the binding NF-κB in the nucleus of T cells, potentially contributing to the lower activation levels of the HIV-1 LTR and possibly promoting viral latency [104]. However, this mechanism has not been explored in cells of the monocyte-macrophage lineage. NF-κB can also function as a repressor of transcription through the recruitment of HDAC1 (Fig. 3) [78,105].

NF-κB DNA binding activity first occurs in monocytes as they progress from promonocytes to monocytes; however, in mature monocytes and MDMs, NF-κB is constitutively active in the nucleus, and its DNA binding activity is not increased further in response to cellular activation or differentiation [106]. This constitutive pool of NF-κB allows a low level of basal HIV transcription in the absence of cellular stimuli. Binding of NF-κB to the enhancer of the HIV-1 LTR plays a critical role in the response of the LTR to cellular stimuli in both T cells and maturing monocytes [36,94,97,106-109]. Deletion or mutation of the NF-κB sites abolishes LTR activity [97,109-112] and results in reduced production of infectious virus [98]. Activation of monocytes by LPS, IL-6, or TNF-α (Fig. 3) results in enhanced HIV replication, a process that correlates with activation of NF-κB [27,49-51,113]. LPS activation of monocytes leads to the induction of the NF-κB pathway through TNF-α [27,50]. In contrast, in differentiated primary MDMs, stimulation by LPS results in the downregulation of LTR activity and viral replication [48]. This activity was not affected by mutation of the NF-κB sites, but did map to the enhancer element (position -156 to -121); thus, this effect may involve NFAT proteins (see below) [48]. While this may seem counter-intuitive, one might speculate that stimulation of cells through the NF-κB pathway would enhance LTR activity and viral replication, it should be noted that LPS stimulation of differentiated macrophages could also induce transcription factors that negatively regulate the LTR, however this has not been explored. This would be very interesting as this might provide another reason for macrophages serving as a latent reservoir for HIV-1. In addition to activating transcription by binding the enhancer region, NF-κB activates transcription by binding to sites -1 to +9 and +31 to +40 relative to the transcriptional start site [114,115].

**Figure 4:**
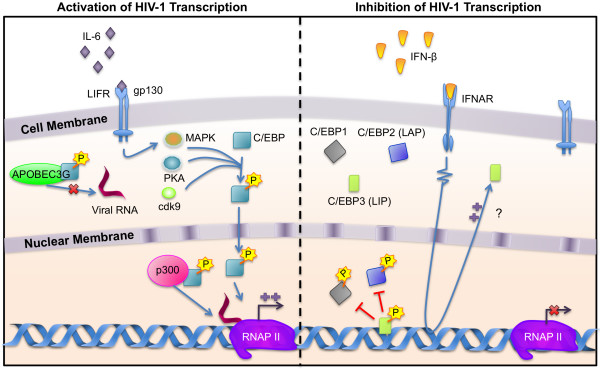
**Important C/EBP transcription factor signaling in monocyte-macrophages**: (a) Activation of HIV transcription: C/EBP, located in the cytoplasm of the cell, can become phosphorylated by the MAP kinase, PKA, or cdk9 through a variety of pathways. Once phosphorylated, C/EBP is translocated into the nucleus where it can transactivate the LTR. In addition, C/EBP associates with histone acetyl transferases such as p300, which when bound to the LTR, make the chromosome accessible for RNA polymerases to bind and transcribe the integrated proviral DNA. Finally, association of C/EBP with APOBEC3G allows for better reverse transcription in the cytoplasm. (b) Inhibition of HIV transcription: The binding of IFNβ to its receptor begins a JAK/STAT signaling cascade that results in increased production of C/EBP3 (LIP). C/EBP3, which does not contain the transactivation domain of full-length C/EBPs, does not interact with histone acetyl transferases and when bound to the LTR, blocks the binding of full-length C/EBPs, thereby leading to a repression of LTR activity.

The NF-κB site(s) located immediately upstream of the Sp sites in the enhancer in HIV and SIV result in Sp-NF-κB protein-protein interactions that further modulate the LTR activity. Sp1 and NF-κB proteins bind the LTR cooperatively and activate transcription synergistically in response to cellular stimulation [66,82,109]. This activation is mediated by the binding of the DNA-binding domain of p65 to the DNA-binding domain of Sp1 [108] (Fig. 4). Sp3 and Sp4 are unable to activate transcription cooperatively with NF-κB [66]. In the absence of functional Sp sites (or in the presence of genetic alterations that inactivate the Sp binding sites), binding of NF-κB to the enhancer can restore replication of the virus in T cells [116-118], perhaps by recruiting Sp to the variant sites.

#### NFAT (AP-3)

NFAT proteins are part of a family of Rel-related transcription factors that become active early after T cell activation and are constitutively in monocytes. NFAT exists as several isoforms (NFAT1, NFAT2/NFATc, and NFAT3-5) that activate a variety of genes in immune and non-immune cell populations [119-121]. Like NF-κB, NFAT contains a DNA-binding domain that is homologous to rel and shuttles between the cytoplasm and nucleus in response to cellular stimuli [122,123]. In the cytoplasm, NFAT is dephosphorylated and translocates to the nucleus where it activates transcription of many genes [124-126] (Fig. 4). NFAT can bind DNA as a high affinity dimer or as a lower affinity monomer [127-129]. NFAT proteins frequently cooperate with other transcription factor families when bound to adjacent sites within a promoter.

An NFAT binding site was identified in the HIV-1 LTR at positions -216 and -254, with a footprint extending from -253 to -215 relative to the transcriptional start site [122,130]. Although this site can bind NFAT *in vitro*, this site was later shown not to be necessary for NFAT-mediated activation of the HIV-1 LTR [131,132]. Instead, NFAT binds the NF-κB binding sites in the enhancer in response to cellular activation in T cells and constitutively in monocytes [110,112,127,130,133]. NFAT activation of genes from κB-like sequences has been documented with a number of host and viral promoters [134,135] (Fig. 4). In addition to binding to the enhancer, NFAT binding at positions +169 to +181 has been reported to activate transcription [59,60,136].

NFAT proteins activate HIV-1 transcription and replication in a variety of cell types. Whereas NFAT1 and NFAT2/NFATc are responsible for the activation of HIV in T cells [110,133,137] reviewed in [138,139]), NFAT5, the most evolutionarily divergent NFAT member, regulates HIV replication in monocyte-MDMs [130]. Terminally differentiated MDMs constitutively express high levels of NFAT5, which is able to bind and activate the enhancer of HIV-1 subtypes B, C, and E, HIV-2, and SIV from multiple primate species [130]. Targeting NFAT5 with siRNAs in primary MDMs modestly reduces viral replication [130]; however, NFAT derived from MDM nuclear extract was unable to compete with NF-κB for binding to the HIV enhancer *in vitro *[98]. This finding suggests that *in vivo*, although constitutively expressed NFAT is able to bind the LTR, it is unable to do so in the presence of high levels of NF-κB.

### Modulatory region

As its name implies, the modulatory region of the LTR functions to regulate transcription that is driven by the core and/or enhancer regions. A wide array of host and viral proteins bind the modulatory region of the LTR to either enhance or repress transcription [45,46,140]. In HIV-1, the loss of both the Sp and NF-κB sites effectively inactivates the LTR. In contrast, the modulatory region of SIVmac and HIV-2 have functional elements that are not present in HIV-1 that can compensate, at least in part, for the loss of the Sp and NF-κB sites [85]. Also, unlike the HIV-1 LTR, the 5' 364 bp of the 517 bp-long U3 region is dispensable for SIV replication [141-143]. Early reports investigating the role of the HIV-1 modulatory region identified bases -423 to -167 as a negative regulatory element (NRE) that repressed LTR activity [144]. Since then, this region has been shown to activate as well as to repress transcription (for review see [140]).

### Basic leucine zipper transactivator proteins

C/EBPs, activating transcription factor/cyclic AMP response element binding (ATF/CREB) proteins, and AP-1 factors are members of a large family of basic leucine zipper (bZIP) proteins that play important roles in the regulation of retroviral transcription [145-147]. Dimerization of the bZIP family members occurs in the C-terminal α-helical leucine zipper domain and is necessary for binding to DNA (reviewed in [148,149]). C/EBP, AP-1, and ATF/CREB proteins each have unique binding sites in the modulatory region of the HIV-1 LTR; however, heterodimerization between C/EBP and ATF/CREB or AP-1 family members has been shown to result in binding to sequences that are different from the consensus sequence for either family of factors [146,150-155]. These sequences are often composed of half of the recognition sequence for each protein in the heterodimer [146,150].

#### C/EBP

**Figure 5:**
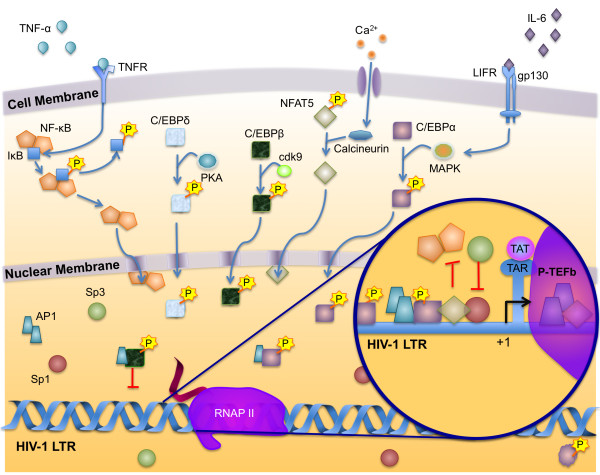
**Regulation of HIV-1 transcription in circulating monocytes**. Transcription of HIV-1 in circulating monocytes is dependent on the ratio of activator to repressor isoforms of transcription factors, the phosphorylation state of transcription factors, and the inducible translocation of NF-κB and NFAT factors from the cytoplasm. NF-κB can be induced to translocate to the nucleus by TNFα-mediated phosphorylation of IκB. NFAT is dephosphorylated in the cytoplasm by calcineurin, which acts in response to calcium levels within the cell. Once it is dephosphorylated, it translocates to the nucleus where it activates transcription by constitutively binding the NF-κB site in the enhancer. Phosphorylation plays a critical role in regulating the activity of C/EBP factors in monocytes. Phosphorylation of C/EBPα by ras-dependent mitogen-activated protein (MAP) kinase, signaled by IL-6 or by cAMP-dependent protein kinase A, results in its nuclear translocation and subsequent transactivation of the LTR. Cyclin-dependent kinase (cdk) 9 specifically phosphorylates C/EBPβ, which then translocates into the nucleus, binds to the LTR, and leads to an increase in HIV-1 gene expression. Once in the nucleus, C/EBP factors then regulate the activity of AP-1 factors. Relatively high levels of C/EBPα dimerize with AP-1 factors to form potent activators of transcription. Lower levels of C/EBPβ balance this activation by binding AP-1 leading to a loss in DNA binding affinity. Sp1 and Sp3 are constitutively expressed in the nucleus. In the presence of Sp1, which is a strong activator, Sp3 competes for binding to the LTR and inhibits activation by Sp1.

The HIV-1 LTR contains three C/EBP binding sites upstream of the transcriptional start site [156,157] and one binding site downstream of the transcriptional start site, at the 3'-most end of U5 (Liu and Wigdahl, unpublished observations). C/EBPs play a critical role in HIV-1 replication. It has been shown that at least one upstream C/EBP binding site and the presence of C/EBP proteins are necessary for replication in cells of the monocyte-macrophage lineage [157-161]. The two C/EBP binding sites located in the U3 region of the LTR have differing affinities for C/EBP factors, with the upstream site (site II), having a much higher relative affinity than the downstream site (site I) [43]. In addition to activating HIV-1 transcription through direct binding to the LTR, C/EBP factors may inhibit the host cellular antiviral protein APOBEC3G (Fig. 5), allowing more efficient reverse transcription to occur in the cytoplasm [162].

The C/EBP family of transcription factors consists of six members, including C/EBP α, β, γ, δ, ε, and ζ [163-169]. C/EBPβ itself has three isoforms that result from the use of internal start codons within a single mRNA [170,171]. C/EBP-1, the full-length isoform, and C/EBP-2, an isoform that lacks the N-terminal 23 amino acids, both contain three transcriptional activation domains and function as activators of HIV-1 transcription. C/EBP-3, which lacks the N-terminal 198 amino acids that include the activation domains, serves as a repressor of HIV-1 transcription, because it retains the C-terminal DNA-binding domain and competes for binding with the activator isoforms of C/EBP.

C/EBP isoform expression depends on the differentiation and activation state of cells in the monocyte-macrophage lineage. C/EBPα levels are high early in monocyte differentiation and then decrease as cells mature, whereas C/EBPβ and C/EBPδ levels are low early in development and increase as cells mature [172,173]. C/EBP isoform expression is also regulated by extracellular stimuli. C/EBPβ expression increases upon cellular activation, whereas expression of the other C/EBP isoforms remains constant [172,174]. Exposure of macrophages to interleukin-1 (IL-1), tumor necrosis factor α (TNFα), or interferon-γ, all of which have been shown to be present at elevated levels during the course of HIV-1 infection, has been shown to induce a reduction in C/EBPα mRNA levels while the levels of C/EBPβ and C/EBPδ expression increase [174]. This results in C/EBPβ and C/EBPδ playing a key role in the regulation of HIV-1 transcription as disease progresses and inflammatory cytokine levels increase (Fig. 4).

An additional level of regulation of C/EBPβ activity resides in two regulatory domains that lie between the activation domains and the DNA binding domain. These domains inhibit C/EBP activity, until phosphorylation results in an increase in DNA binding affinity and transcriptional activation activity [175,176]. Several signaling cascades regulate the phosphorylation state of C/EBP. Phosphorylation of threonine 235 by a ras-dependent mitogen-activated protein kinase increases transcriptional activation [177]; phosphorylation of serine 288 by cAMP-dependent protein kinase A results in nuclear translocation and subsequent transactivation [178]; and cyclin-dependent kinase 9 (cdk9) phosphorylates C/EBPβ and leads to an increase in HIV-1 gene expression [179] (Fig. 4).

C/EBPs interact with many nuclear proteins to activate transcription. In addition to binding other bZIP proteins, C/EBP recruits chromatin remodeling complexes such as SWI/SNF[180], cAMP response element-binding protein/p300 [181,182], and p300/CREB-binding protein-associated factor [183] to the HIV-1 LTR. These proteins remodel the chromatin structure and increase transcription of the HIV-1 genome. C/EBP increases the phosphorylation of p300, which in turn alters its nuclear localization and increases its activity [184]. C/EBP can also act synergistically with Sp proteins to activate transcription of the HIV-1 LTR [185].

The importance of C/EBP factors in the regulation of HIV-1 gene expression is underscored by the discovery that a 6G configuration (a T-to-G change at nucleotide position 6) in C/EBP site I increases C/EBP binding, increases LTR activity, and is preferentially encountered in proviral LTRs derived from the brain of HIV-1-infected patients [42,186]. C/EBP site II was also found to be preferentially conserved in the consensus subtype B configuration or to contain a 6G variation of this site, which are both high affinity sites for C/EBP factors in LTRs present in proviral DNA in cells located in the mid-frontal gyrus of the brain of infected individuals. A high rate of viral replication occurs in this region of the brain. Interestingly, the presence of the 6G configuration of this binding site also correlates with the presence of HIV-1-associated dementia [42,44]. In contrast, the presence of a 4C C/EBP site II, which is a low-affinity C/EBP site, has been found preferentially in the cerebellum, a region of low viral replication [44]. This observation suggests that high affinity for C/EBP factors may contribute to the maintenance and/or pathogenesis of HIV-1 in the central nervous system, whereas low affinity sites such as 4C may contribute to lower levels of transcription required to maintain a latent reservoir of provirus. We have also identified a 3T configuration (a C-to-T change at position 3) of C/EBP site I that exhibits a low affinity for C/EBP within LTRs in the peripheral blood and brain and has also been shown to correlate with both late stage HIV disease and HIV-1-associated dementia [43], respectively.

#### ATF/CREB

ATF/CREB binds the HIV-1 LTR at a site immediately upstream of the C/EBP binding site I [38,187] and at two sites downstream of the transcriptional start site (sites +160 to +167 and +92 to +102) to regulate the LTR [59,60,188,189]. ATF/CREB and C/EBP factors can bind their adjacent upstream sites individually as homodimers, or C/EBP and ATF/CREB can heterodimerize with each other to regulate HIV-1 expression. This heterodimerization results in the recognition of a site composed of the 3' half of the ATF/CREB site and the 5' half of the C/EBP site [146]. As a result, in the presence of genetic variation that results in a low affinity C/EBP site, ATF/CREB is able to recruit C/EBP factors to the site and vice versa [146]. In addition to activating transcription, ATF/CREB can inhibit transcription by binding to Swi6, a component of the remodeling complex SWI/SNF, to promote the formation of heterochromatin [190].

#### AP-1 (Fos/Jun)

AP-1 proteins exist as homodimers of Jun family members (c-Jun, JunB, and JunD) or as heterodimers of Jun and Fos family members (c-Fos, FosB, Fra-1, and Fra-2) (reviewed in [191]). They bind a palindromic DNA sequence known as the TPA-responsive elements (TRE) at positions -306 to -285 and -242 to -222 of the LTR [59] as well as at positions +95 and +160, downstream of the transcriptional start site [59,60,188,189]. The sequence of these sites has been shown to evolve in a manner that facilitates efficient cell type-specific binding of AP-1 [59,192]. AP-1 acts as either an activator or repressor of transcription, depending on the components of the dimer [191,193]. Once bound to the promoter, cFos/cJun heterodimers can recruit the SWI/SNF chromatin remodeling complex to activate transcription, whereas homodimers or heterodimers consisting of other family members lack this ability [194].

AP-1 mRNA is typically absent in quiescent cells; however, it is significantly up-regulated upon cellular stimulation [195]. Jun levels increase during monocytic maturation and become constitutively expressed in MDMs [196-200]. Despite being expressed, AP-1 in MDMs of some tissues, such as the lung, lacks the ability to bind DNA because of the lack of expression of Ref-1, a protein that modulates the oxidation state of Fos [201,202]. In addition to being regulated by oxidation [201-203], AP-1 protein activity is further controlled post-transcriptionally by sumoylation, which inhibits protein activity [204,205], and by phosphorylation, which increases activity in response to cellular stimulation [206].

In addition to directly regulating HIV-1 gene expression, AP-1 proteins can modulate the activity of other transcription factors. C/EBPβ dimerization with c-Fos or c-Jun results in C/EBP being unable to bind DNA thus a reduction in C/EBP-mediated transactivation [153,154]. In contrast, C/EBPα dimerization with c-Jun or c-Fos forms a potent activator of transcription [207]. In response to mitogen or cytokine stimulation, the mitogen-activated protein kinases ERK1/ERK2 phosphorylate AP-1 (reviewed in [208] and [209]). This phosphorylation promotes the interaction of AP-1 with NF-κB and the enhancer element, which leads to the synergistic activation of the LTR [210-213]. This cascade of events is one mechanism by which HIV emerges from latency [210,214].

#### Tat

Tat is a virus-encoded transcriptional transactivator that binds to the RNA secondary structure encoded by the transactivation region (TAR) in the repeat segment of the LTR (+1 to +59) [215,216]. Once bound to the elongating transcript, Tat helps assemble the pre-initiation complex and recruits cdk9 to promote phosphorylation of RNA Pol II [217,218] and P-TEFb to increase processivity of RNA Pol II [219-223]. Interestingly, mechanistic studies of this complex suggest that one of the functions of Tat is to increase the duration of P-TEFb occupancy at the HIV-1 LTR [224]. Tat also significantly remodels chromatin by recruiting the histone acyltransferases Tip60 [225,226], human Nucleosome Assembly Protein-1 (hNAP-1) [227], p300/cAMP response element-binding protein [228,229], and p/CAF [230], as well as the chromatin-remodeling complex SWI/SNF [231]. Tat activity is limited in monocytes due to the lack of sufficient levels of cyclin T1, a component of P-TEFb [54]. Differentiation into macrophages increases Cyclin T1 expression and results in strong Tat activity [54].

Tat regulates the activity of many other transcription factors through direct protein-protein interactions and the modulation of kinase activities. Tat promotes the phosphorylation of Sp1, which in turn increases binding of Sp to the LTR [92]. Conversely, Sp is also necessary to recruit Tat to the LTR [76], and deletion or mutation of the Sp binding sites in the promoter abolishes Tat activity [232,233]. It is currently unclear whether direct interaction occurs between Sp factors and Tat [234-236]. In addition to regulating Sp1 activity, Tat increases the cooperation between NFAT and AP-1 proteins without altering independent binding of these transcription factors to DNA [137,237]. It also promotes the interaction of NF-κB and AP-1 factors to synergistically activate transcription [238-240].

#### Vpr

Vpr is another virus-encoded protein that plays a direct role in the regulation of HIV-1 transcription [241-243]. Vpr is found in the viral particle and plays an important role in early transcriptional activation of the LTR before Tat can be expressed [244-248]. Its importance is highlighted by a recent study that describes alterations in Vpr that provide a significant reduction in Vpr nuclear import and virion incorporation uniquely in a long term non-progressor patient [249]. Vpr also causes cell cycle arrest in the G2 phase, the phase of the cell cycle when the LTR is most active, which results in apoptosis. [250]. It is necessary for viral replication in cells of the monocyte-macrophage lineage [251-256]. Interestingly, Vpr has been shown to interact with the nuclear form of uracil DNA glycosylase (UNG2), a cellular DNA repair enzyme, which helps incorporate this protein into virus particles leading to a decrease in viral mutation rate. Specifically, the lack of UNG in virions during virus replication in primary monocyte-derived macrophages further increases virus mutant frequencies by 18-fold compared with the 4-fold increase measured in actively dividing cells [257]. In addition, Vpr has been shown to concentrate at the nuclear envelope (NE) shortly after infection (4-6 hrs) as part of the pre-integration complex (PIC), supporting an interaction between Vpr and components of the nuclear pore complex [258-261], including the nucleoporin hCG1 [262]. Single-point Vpr mutants within the first α-helix of the protein such as Vpr-L23F and Vpr-K27M fail to associate with hCG1, but are still able to interact with other known relevant host partners of Vpr. In primary human monocyte-derived macrophages, these mutants fail to localize at the NE resulting in a diffuse nucleocytoplasmic distribution, impaired the Vpr-mediated G2-arrest of the cell cycle, and subsequently induced cell death. These observations were obtained in primary macrophages from some but not all donors indicating that the targeting of Vpr to the nuclear pore complex may constitute an early step toward Vpr-induced G2-arrest and subsequent apoptosis. These results also suggest that Vpr targeting to the nuclear pore complex is not absolutely required, but can enhance HIV-1 replication in macrophages [263]. Extracellular Vpr is found in the plasma and the CSF [254,264] and can enter monocytes and macrophages and behave as if the protein was endogenously expressed [265-267]. Vpr binds the LTR in a sequence-specific manner to activate transcription directly [45,46] and also interacts with Sp1 [268], TFIIB [269,270], NF-κB [271], C/EBP [272], and Tat [244,273] to enhance transcription of the HIV-1 genome. Vpr activates the DNA binding activity of AP-1 by promoting the phosphorylation of cFos and cJun in monocytes and macrophages [267]. It also promotes the translocation of NF-κB p50/p65 to the nucleus by promoting the phosphorylation of IκB [267], which allows an NF-κB- and AP-1-mediated increase in LTR activity.

C/EBP and Vpr interact at the HIV-1 LTR in two ways. Vpr has been shown to increase C/EBPβ DNA binding activity [272]. It has also been shown that Vpr has a high affinity for LTR C/EBP binding site I variants that exhibit a decreased affinity of the site for C/EBP. The presence of these LTR variants correlates with late-stage HIV-associated disease [45,46]. Thus, as HIV-1-associated disease progresses, viral variants containing this type of LTR C/EBP site I may become more prevalent and function to facilitate a transition from C/EBP-mediated LTR activation to Vpr-mediated transactivation from that site. Alternatively, Vpr and C/EBP may form a complex at that site (Burdo and Wigdahl, unpublished observations). In addition to interacting with cellular proteins, Vpr interacts with Tat and activates transcription in an additive manner [244,274].

#### Methylation

HIV proviral DNA that has integrated into the host genome also becomes subject to host factors that regulate chromatin organization and gene transcription. These mechanisms include histone modification, RNA interference/silencing, and DNA methylation. The mechanisms play a role in the control of gene expression, viral activation, and/or latency. DNA methylation of CpG islands within the HIV-1 LTR is one process that results in the downregulation/silencing of the integrated proviral genome [275-278]. This form of transcriptional silencing occurs by specific methyltransferases that are directed to the target DNA by methylation of lysine 9 of histone H3 through histone methyltransferases [279]. In cells of the monocyte-macrophage lineage, methylation of the LTR has been found to result in the transcriptional silencing of the promoter which contributes to limited access of transcription factors to the target DNA [280]. In addition, in the CD4^+ ^T cell line ACH-2, the transcriptional silencing brought about by DNA methylation of the LTR can be reversed through TNF-α treatment of the cells which leads to demethylation of the 5' LTR and the induction of viral gene expression [281] showing that although this modification is inheritable, it is not permanent. The reduction of LTR expression is possibly explained by the binding of methyl-CpG-binding protein 1 complex and methyl-CpG-binding protein 2 to methylated Sp1 transcription factor binding sites, thereby inhibiting the binding of Sp1 transcription factors [282,283]. In addition, the transcription factors USF and NF-κB lose affinity for their methylated LTR transcription factor binding sites as well [284]. Unfortunately, to date all of these studies have been performed in T cell lines and primary T cells, but not in cells of the monocyte-macrophage lineage.

#### Cytokines

Cytokines play a critical role in the pathogenesis of HIV-1. IL-6, TNFα, IL-1β, and other proinflammatory cytokine levels are elevated in the blood, bone marrow, and cerebrospinal fluid of HIV-infected patients [285,286]. IL-6 and TNF-α are induced early after HIV monocytic infection, followed by their continued increased expression [52,53,287]. IL-6 is a potent activator of C/EBP, and exposure of monocytes to IL-6 results in increased HIV-1 replication. The increase in C/EBP activity then forms a positive feedback loop for IL-6 expression, because C/EBPβ binds to and activates the IL-6 promoter [288]. C/EBPs can also activate the genes encoding other proinflammatory cytokines such as IL-1β [289] and TNFα [290,291]. TNFα is one of the most potent activators of NF-κB activity known. It acts by causing a signaling cascade that activates the IκB kinase complex, which then phosphorylates IκB, releasing NF-κB. The free NF-κB translocates to the nucleus and induces the activation of the HIV-1 LTR (Fig. 3 and 4).

In addition to being regulated by cytokines, chemokines contribute to HIV-1 infection and pathogenesis. The HIV-1 Nef protein induces HIV-infected macrophages to secrete at least two chemokines, MIP1α and MIP1β, which recruit and activate resting CD4+ T lymphocytes [292]. These T cells can then become infected and produce high levels of virus.

### Summary of important monocytic regulatory pathways regulating the HIV-1 LTR

Regulation of HIV-1 transcription in cells of the monocyte-macrophage lineage varies considerably with the stage of cellular differentiation as well as in comparison to activated T cells. Specifically, it has been observed that cyclin T1 expression in monocytes is controlled by differentiation. Cyclin T1 increases as cells of the monocyte-macrophage lineage differentiate [47]. Unstimulated peripheral blood monocytes and myeloid progenitor cells support low levels of viral replication and activate transcription in response to cellular activation like T cells [27,36,48-54] whereas differentiated MDMs have increased viral replication but either do not respond to [45] or downregulate HIV transcription [48,55] in response to cellular stimulation. As cells of the monocyte lineage differentiate, the ratio of Sp1 to Sp3 increases, resulting in an increase in HIV-1 transcription (McAllister and Wigdahl, unpublished observations). This process may result in low level HIV replication, or viral genomic silence, in circulating monocytes, and evasion of the host immune system until the cells are differentiated in peripheral tissues. The importance of the Sp sites also varies depending on the differentiation stage of the cell; in unstimulated monocytes, mutation of the Sp sites reduces LTR activity, whereas in MDMs, transcription of HIV and replication of SIVmac are abolished when these critical binding sites are knocked out [83-86]. NF-κB regulation of the LTR is also unique in MDMs. In MDMs, NF-κB is composed of Rel B bound to p50 or p52, whereas NF-κB in T cells is predominantly composed of p65 or c-Rel bound to p50 or p52 [97-100]. NF-κB DNA binding activity first occurs in monocytes as they progress from promonocytes to monocytes; however, in mature monocytes and MDMs, NF-κB is constitutively active in the nucleus, and its DNA binding activity is not increased further in response to cellular activation or differentiation [106]. Stimulation of T cells and monocytes by LPS results in enhanced HIV replication, a process that correlates with activation of NF-κB [27,49-51,113]. In differentiated primary MDMs, stimulation by LPS results, however, in the downregulation of LTR activity and viral replication [48].

NFAT, C/EBP, Jun and AP-1 transcription factor regulation of LTR activity also have distinct differences in monocyte-macrophages compared to T cells. NFAT binds the NF-κB binding sites in the enhancer in response to cellular activation in T cells but binds constitutively in monocytes [110,112,127,130,133]. Also, NFAT5, the most evolutionarily divergent NFAT member, regulates HIV replication in monocyte-MDMs [130] but has not been shown to do this in T cells. With regard to C/EBP, it has been shown that at least one upstream C/EBP binding site and the presence of C/EBP proteins are necessary for replication in cells of the monocyte-macrophage lineage but not in T cells [157-161]. Jun levels increase during monocytic maturation and become constitutively expressed in MDMs [196-200]. Despite being expressed, AP-1 in MDMs of some tissues, such as the lung, lacks the ability to bind DNA because of the lack of expression of Ref-1, a protein that modulates the oxidation state of Fos [201,202].

The viral proteins Tat and Vpr have also been shown to have unique properties with regard to HIV-1 LTR activation in cells of the monocyte-macrophage lineage. Tat activity has been shown to be limited in monocytes due to the lack of sufficient levels of cyclin T1, a component of P-TEFb [54]. Differentiation into macrophages increases Cyclin T1 expression and results in strong Tat activity [54]. Vpr has been shown to be necessary for viral replication in cells of the monocyte-macrophage lineage but not in T cells [251-256]. Vpr has also been shown to specifically play a role in viral mutation rates in cells of the monocyte-macrophage lineage. Specifically, the lack of UNG in virions due to lack of Vpr binding to UNG during viral packaging led to increased virus mutant frequencies as indicated previously (18-fold increase compared to a 4-fold increase) [257]. In addition, genetic variation in Vpr has been shown in primary human monocyte-derived macrophages to fail in Vpr localization at the NE resulting in a diffuse nucleocytoplasmic distribution, impairing the Vpr-mediated G2-arrest of the cell cycle and the subsequent cell death induction, in some but not all donors [263].

## Conclusions

Regulation of HIV-1 transcription in cells of the monocyte-macrophage lineage is a complex process involving the interaction of numerous factors that are expressed in a differentiation-dependent manner and whose activity is regulated by both cellular differentiation and extracellular signaling pathways. Although monocytes can be infected, this process is hindered at multiple steps in the viral lifecycle, including transcription. The mechanism behind the block to replication in monocytes has yet to be fully characterized, but it is clear that many factors make contributions. Monocytes express relatively low levels of the HIV co-receptor CCR5 [293,294] and recently, it has been shown that viral entry is impaired in circulating monocytes [295]. Reverse transcription and integration are also impaired [295,296]. At the transcriptional level, LTR activity is regulated by the ratio of activator to repressor isoforms of transcription factors, the phosphorylation state of transcription factors, the inducible translocation of NF-κB and NFAT factors from the cytoplasm, and the availability of viral transactivator proteins and their host co-factors (Fig. 4). Members of the AP-1 transcription factor family and relatively equal levels of nuclear Sp1 to Sp3 facilitate a modest level of basal transcription, whereas NF-κB and NFAT proteins remain sequestered in the cytoplasm in the early stages of monocytic differentiation. The presence of Tat has little effect on transcription in monocytes, as cyclin T1 expression is undetectable and other factors required for Tat activation are absent [54]. This lack of Tat activity contributes to replication block observed in unstimulated circulating monocytes.

**Figure 6:**
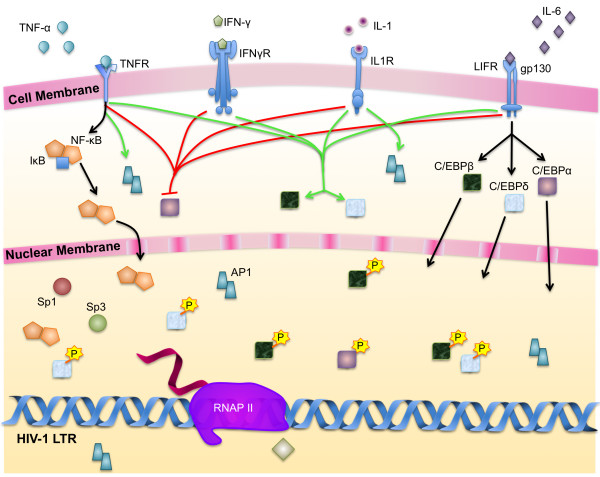
**Cytokine-regulation of HIV-1 transcription in monocytes**. Cytokines play an integral role in regulating the availability and activity of transcription factors that regulate the LTR. TNFα strongly induces the nuclear localization of NF-κB in monocytes. As a result, the subsequently stimulated LTR interfaces with increased levels of Sp and NF-κB factors. Cellular activation increases the expression of C/EBP, particularly activation by IL-6. TNF-α, IL-1, and interferon-γ reduce the expression of C/EBPα and increase expression of both C/EBPβ and C/EBPδ. Stimulation increases the expression of AP-1 in the cell where its interaction with NF-κB at the enhancer element leads to synergistic activation of the LTR. (**Black arrows**: translocation to nucleus; **red arrows: **decrease in expression; **green arrows**: increase in expression).

Although circulating monocytes exhibit low levels of viral replication, replication increases in response to cytokine stimulation. During periods of inflammation caused by HIV-1 infection, co-pathogens, or opportunistic infections, levels of circulating cytokines such as IL-6 and TNFα increase and stimulate HIV-1 replication in monocytic cells. IL-6 increases the activity of C/EBP factors; these factors then activate the LTR and form a positive feedback loop by activating the promoters of cytokines, including TNFα and IL-6. In response to TNFα, NF-κB and NFAT5 translocate to the nucleus, and AP-1 DNA binding activity is stimulated to activate transcription as a result of changes in protein phosphorylation (Fig. 6). NFAT and NF-κB interact at the enhancer to activate transcription synergistically, whereas ATF/CREB, AP-1, and C/EBPα, β, and δ form homo- and heterodimers to regulate LTR activity. Vpr binds to the LTR directly and through interactions with other factors associated with the transcriptional complex in conjunction with AP-1, NF-κB, and C/EBP to activate transcription.

**Figure 7:**
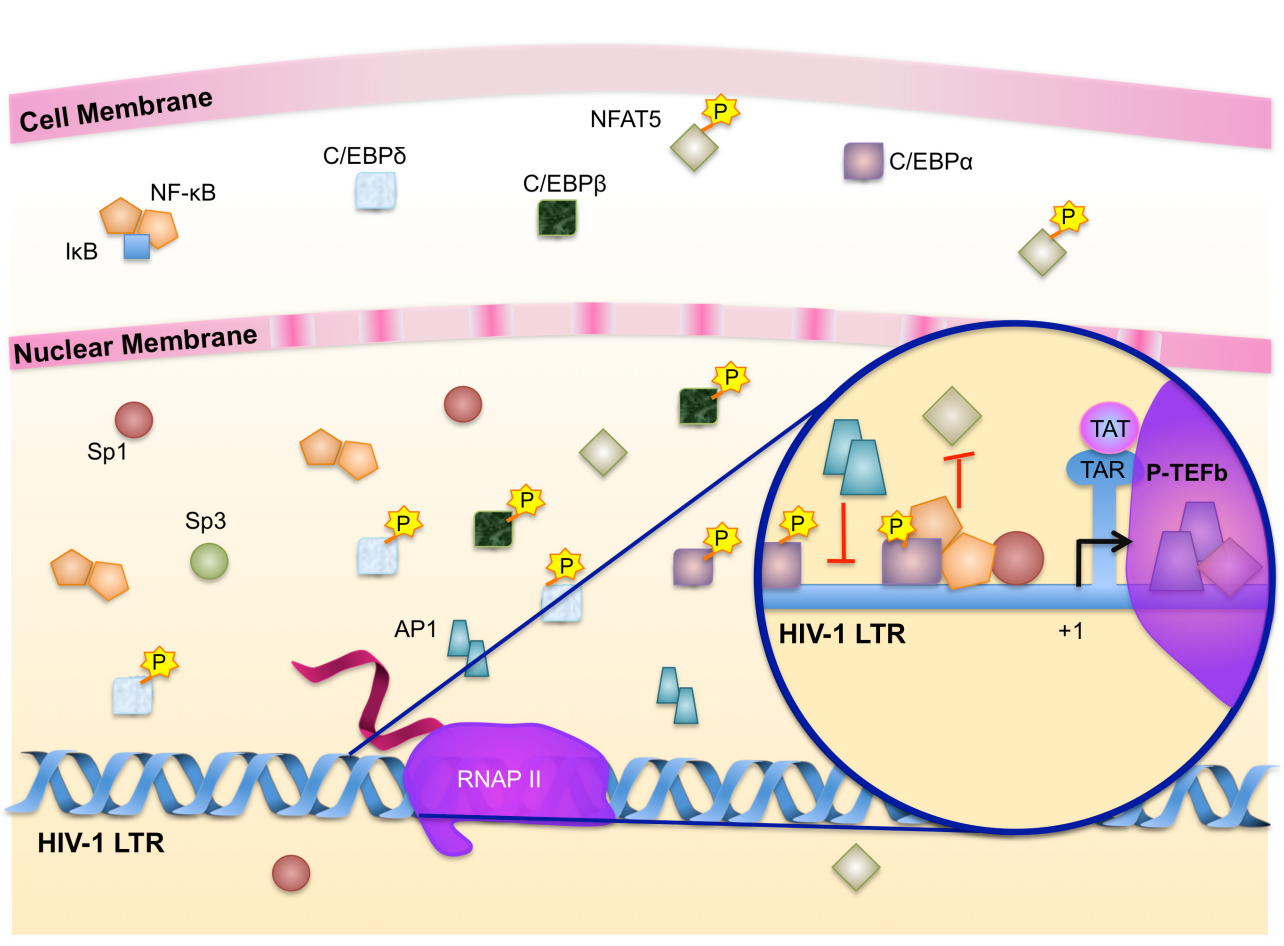
**Regulation of HIV-1 transcription in differentiated macrophages**. In differentiated macrophages, NF-κB and NFAT are constitutively localized in the nucleus; however, in the presence of large amounts of NF-κB, NFAT is unable to bind the LTR. NF-κB-Sp1 protein-protein interactions bind the LTR cooperatively and activate transcription synergistically in response to cellular stimuli. Sp sites are necessary for viral replication, and the ratio of Sp1 proteins to Sp3 proteins increases, thus increasing transcription of the virus. As the cell matures, C/EBPα levels decrease and C/EBPβ and C/EBPδ levels increase. AP-1 is constitutively expressed but loses its ability to bind to the LTR. Tat binds to the transactivation response region (TAR) structure on the viral RNA and recruits (P-TEFb (the Cyclin dependent kinase 9 (Cdk9) and cyclin T1 (CycT1) complex) through binding to cyclin T1. Recruitment of P-TEFb to TAR induces hyperphosphorylation of CTD by Cdk9, thereby enhancing the transcriptional elongation of HIV-1.

As monocytes differentiate into macrophages, the permissiveness to viral replication increases dramatically, although MDMs lose the ability to further increase viral replication in response to extracellular stimuli. Cofactors necessary for Tat transactivation of the LTR are expressed, allowing a much greater level of HIV-1 transcription than is possible in monocytes. NF-κB, AP-1, and NFAT proteins are constitutively localized in the nucleus, and the activator Sp1 expression predominates over the repressor Sp3, resulting in greater availability of Sp1 (Fig. 7). The viral protein Nef also activates signaling cascades that result in enhanced binding of AP-1 to the LTR and enhanced cooperation between AP-1 and NF-κB [210,214].

Although macrophages support active viral replication, they are recognized as reservoirs of HIV-1 and quietly harbor the virus during latency. Host proteins that contribute to LTR activation in macrophages during productive viral infection ironically may also contribute to transcriptional silencing during latency. Sp1 proteins have been shown to bind the LTR constitutively, regardless of the level of transcription [297], and, in the latent stage, Sp1, NF-κB, AP1, and ATF/CREB may function as repressors of transcription by recruiting HDACs to the LTR and promoting the formation of heterochromatin. Although AP-1 proteins become constitutively expressed, the level of Ref-1, which is required for the DNA binding activity of AP-1, is significantly reduced in the nucleus of MDMs [201,202]. This effectively renders nuclear AP-1 proteins inactive. In addition to their inability to transactivate the LTR, the constitutive presence of AP-1 proteins may be sufficient to disrupt the binding of C/EBP to the LTR, because inactive heterodimers of AP-1 and C/EBP may be more likely to form in the presence of excess AP-1 proteins. It is currently unknown what triggers the switch from latency to productive replication, however the presence of factors that can serve as both activators and repressors at the LTR likely contributes to the ability of the virus to resume replication very quickly upon the removal of repressive stimuli such as HAART therapy.

Genetic variation within the LTR also plays a role in HIV-1 transcription as HIV-associated disease progresses. Previous studies have shown that Vpr binds with high affinity to specific configurations of sequences within the HIV-1 LTR C/EBP site I and NF-κB site II, and may directly activate transcription. The HIV-1 LTR C/EBP-NF-κB genotypic configuration that exhibits high affinity for Vpr and low affinity for C/EBPβ is prevalent during late stage HIV/AIDS and in LTRs preferentially encountered in autopsied brain tissue from individuals with HAD at the time of death as compared to that from individuals without HAD. In parallel with these observations, additional studies have identified specific variants of the viral transactivator Tat from HAD brain tissue that are defective with respect to their ability to transactivate the LTR, but still retain the ability to activate promoters of a number of proinflammatory cytokine genes [298]. In some tissues, such as the brain, Tat may become less transcriptionally competent as HIV-associated disease progresses. In these circumstances, it is postulated that Vpr facilitates HIV-1 replication by transactivation of LTR-directed transcription in the absence of a fully active Tat protein.

### Future directions

Transcription of the HIV-1 LTR is a highly complex process that involves the interplay of host and viral transcription factors coupled with a wide array of signaling pathways that are activated by extracellular stimuli. Targeting transcriptional pathways in drug discovery recently proved effective in treating certain cancers and may provide an opportunity for additional therapeutic agents in the highly active retroviral therapy (HAART) repertoire. Stat3 has been declared "one of the most important oncogenic transcription factors against which a targeted therapy is needed" [299]. Constitutive Stat3 activity has been observed in many cancers, including prostate [300], squamous cell [301], breast [302,303], head, and neck cancers [302], and has been associated with a poor prognosis [300]. c-Myc activity has been implicated in prostate cancer, melanoma, and Burkitt's lymphoma, and an anti-myc antisense oligonucleotide has made it to clinical trials for the treatment of prostate cancer [304]. Transcription factors that play critical roles in the regulation of HIV-1, including NF-κB and Sp factors, are also the target of anti-cancer drug development. NF-κB has been implicated in playing a role in tumorigenesis in a variety of cancers [305,306], including colon [307], prostate, breast, and lung [308,309]. Small molecule inhibitors that target NF-κB are currently under development for the treatment of cancers [305], and have shown promise in small animal models [310,311]. Many of these inhibit IκB phosphorylation, resulting in NF-κB being sequestered in the cytoplasm [312]. Bortezomib was recently approved by the FDA for the treatment of multiple myeloma. Developed as a reversible 26S proteasome inhibitor, it is now believed that its antitumor activity may be attributable to its inhibition of NF-κB [313-315]. Tolfenamic acid, a nonsteroidal anti-inflammatory drug approved for the treatment of migraine headaches, has been shown to inhibit pancreatic cancer cell growth in vitro and pancreatic and esophageal tumor growth in vivo by inducting the proteosomal degradation of Sp factors [316-320]. It also has been shown to decrease AP-2 and YY-1 transcription factor expression in breast cancer cells and tumors [320]. P-TEFb has been a target of chemotherapies for the treatment of renal, gastric, and lung cancers, as well as mantle-cell lymphoma, however clinical trials revealed that drugs targeting this factor were not effective as monotherapies but showed some promise when combined with other treatments [321-325]. Drugs targeting P-TEFb have been shown to inhibit HIV-1 transcription and replication in a dose-dependent manner in cell lines with minimal cytotoxicity, however the drugs were less effective and more cytotoxic in primary PBMCs [326]. Further study is necessary to determine the feasibility of applying other chemotherapeutic drugs that target host transcription factors to HAART therapy with important components of the developmental pathway focused on minimizing toxicity.

Vpr and Tat provide obvious candidates for targeted drug therapy directed against HIV. Inhibition of Vpr-mediated nuclear import by the compound hematoxylin has been shown to decrease viral replication [327], and fumagillin has been shown to suppress HIV-1 infection of macrophages by targeting Vpr-mediated growth arrest and transcriptional activity [328]. Peptide analogs of Tat have been shown to inhibit Tat's ability to recruit cdk2 to the LTR, and to decrease transcription in vitro and viral load in a small animal model of HIV-1 infection [329]. Small molecular inhibitors have also been developed that disrupt the Tat-TAR interaction, however these have not developed into clinical trials [330-333]. In addition to targeting individual viral proteins, unique structural motifs created at the interface between these factors and host transcription factors should also be considered in future studies.

## Abbreviations

AIDS: acquired immunodeficiency syndrome; AP-1: activator protein 1; ATF/CREB: activating transcription factor/cyclic AMP response element-binding; bZIP: basic leucine zipper; C/EBP: CCAAT enhancer binding protein; cdk9: cyclin-dependent kinase 9; HAD: HIV-1-associated dementia; HDACs: histone deacetylases; HIV: human immunodeficiency virus; HIV-1: human immunodeficiency virus type 1; HIV-2: human immunodeficiency virus type 2; IL-1: interleukin-1; LTR: long terminal repeat; MDMs: monocyte-derived macrophages; NFAT: nuclear factor of activated T cells; NF-κB: nuclear factor kappa B; NRE: negative regulatory element; SIV: simian immunodeficiency virus; Sp: stimulatory protein; TNFα: tumor necrosis factor α.

## Competing interests

The authors declare that they have no competing interests.

## Authors' contributions

EK was responsible for drafting and revising the manuscript as well as organizing the content. SS created Figures 1, 2, 3, 4, 5, 6, 7 and their legends and proofread the final version of the manuscript for content and consistency. MN drafted portions of the manuscript, assisted in the conceptualization of the figures, and proofread and edited the final version of the manuscript. BW assisted in all aspects of each phase of development from initial concept, through revisions to final approval of the version to be published.
